# Presentations to an urban emergency department in Bern, Switzerland associated with acute recreational drug toxicity

**DOI:** 10.1186/s13049-017-0369-x

**Published:** 2017-03-07

**Authors:** Evangelia Liakoni, Sabine Müller, Adrian Stoller, Meret Ricklin, Matthias E. Liechti, Aristomenis K. Exadaktylos

**Affiliations:** 1 Department of Nephrology, Hypertension and Clinical Pharmacology, Inselspital, Bern University Hospital, University of Bern, Bern, Switzerland; 2 Division of Clinical Pharmacology and Toxicology, Basel University Hospital and University of Basel, Basel, Switzerland; 3 Emergency Department, Inselspital, Bern University Hospital, University of Bern, Bern, Switzerland

**Keywords:** Recreational drugs, Acute toxicity, Psychoactive substances, Prescription drug abuse, Emergency room

## Abstract

**Background:**

Although the recreational use of psychoactive substances is common there is only limited systematic collection of data on acute drug toxicity or hospital presentations. Currently, data from Switzerland are only available from the University Hospital of Basel. The present study aimed to describe the presentations due to recreational drug use at an emergency department in Bern, Switzerland during a 4 year period.

**Methods:**

Retrospective analysis of cases presenting from May 2012 to April 2016 at the emergency department of the University Hospital of Bern, Switzerland, with symptoms/signs consistent with acute toxicity of recreational drug use. The cases were retrieved using a comprehensive full-text search algorithm of the electronic health records. Isolated ethanol intoxications were excluded.

**Results:**

During the study period, 503 of the 157,328 emergency department attendances were directly related to acute toxicity of substances used recreationally. The mean patient age was 33 years (range 16–74), 68% were male. Alcohol co-ingestion was reported in 54% of the cases, and use of more than one recreational drug in 37% of the cases. Most presentations were related to cocaine (29%), cannabis (26%), heroin (20%) and benzodiazepines/sedatives (18%). Urine drug screening immunoassay was available in 277 cases (55%). The most frequently detected substances were cannabis (29%), cocaine (22%), benzodiazepines (21%) and opioids excluding methadone (20%). There were only two intoxications with novel psychoactive substances (NPSs): One with methylone and one with 2,5-dimethoxy-4(n)-propylphenethylamine (2C-P). The majority of patients (58%) displayed impaired consciousness (Glasgow Coma Scale (GCS) <15) upon presentation and/or pre-hospital; 21% were unconscious (GCS <8). Other frequent symptoms were agitation (36%), tachycardia (29%), and anxiety (24%). Severe complications included two fatalities, three acute myocardial infarctions, two intracranial haemorrhages, as well as psychosis and seizures in 71 and 26 cases, respectively.

**Conclusions:**

Most medical problems related to recreational drug use were associated with cocaine and cannabis use and were mainly characterised by central nervous system depression, sympathomimetic toxicity and/or psychiatric disorders. Presentations related to acute toxicities of NPSs appear to be uncommon, while prescription drugs were after classical recreational drugs the substances most commonly reported.

**Electronic supplementary material:**

The online version of this article (doi:10.1186/s13049-017-0369-x) contains supplementary material, which is available to authorized users.

## Background

The recreational use of psychoactive substances is common. It is estimated that almost a quarter of the adult population in the European Union have tried illicit drugs at some point in their lives [1]. The most commonly used drug in Europe is cannabis, followed by cocaine, 3,4-methylenedioxymethamphetamine (MDMA), and amphetamines [1]. However, levels of lifetime use differ considerably between countries [1]. Substance use data are usually collected on the basis of indicators such as custom seizures, drug-related deaths, and user surveys. However, such data often lack information on the acute toxicities of these substances. Novel psychoactive substances (NPSs, also known as “designer drugs” and “legal highs”) are usually analogues or derivatives of controlled substances produced in order to circumvent regulations and their use has rapidly increased in recent years [2]. In 2015, 98 NPSs were reported to the European Monitoring Centre for Drugs and Drug Addiction (EMCDDA) for the first time, bringing the number of NPSs detected for the first time in the last 5 years to 380 (101 in 2014, 81 in 2013) [1]. The NPSs are typically not detectable with the usual drug of abuse immunoassays. Thus, they can cause acute toxicities and medical complications, including deaths, but escape detection.

There is currently only limited systematic data on acute drug toxicity causing hospital presentations. Well-organised monitoring systems for drug-related health emergencies can expand the limited data currently available on acute toxicities of recreational substances in general and on NPSs in particular, thus contributing to the prevention of medical emergencies and the improvement of management strategies of acute drug toxicity [3, 4]. Data from emergency departments (EDs) provide a unique insight into acute health harms related to drug use and serve both as drug-trend monitoring and early warning tool. Important data in this field come from the European Drug Emergencies Network (Euro-DEN), established in 2013. The project aims to improve knowledge on acute recreational drug toxicity by collecting data on ED visits across Europe [3]. First results showed that opioids were the drugs most frequently associated with acute drug toxicity presentations, with heroin being most commonly involved (24% of the presentations) [5]. Cocaine and cannabis were also prominent (16% each), while NPSs were less commonly reported (11% of the presentations). The most frequent NPSs were cathinones, particularly mephedrone. Currently, data from Switzerland are only available from the University Hospital of Basel [6, 7], where most medical problems related to recreational drug use were associated with cocaine and cannabis and mainly included sympathomimetic toxicity and/or psychiatric disorders. NPSs were infrequently associated with ED presentations, with only four cases in a 2-year period.

As there are no data available from other parts of Switzerland besides Basel [6, 7] and data from only one region are not representative of the country, the present study aimed to describe the presentations related to acute recreational drug toxicity at a large urban ED in another main city of Switzerland, Bern, over a period of 4 years. Our main objective was to collect systematic data on the frequency, type and severity of acute medical problems due to recreational drug use. Furthermore, we compared our findings with those from Basel and from other parts of Europe in order to identify possible local trends and differences.

## Methods

This retrospective study was approved by the local ethics committee (No. 2016–00413). All patients admitted to the ED at the University Hospital of Bern with acute recreational drug-related problems between May 2012 and April 2016 were included. The ED of the Inselspital, University Hospital of Bern, serves as catchment area for about 2 million people in the Canton of Bern, with about 40,000 emergency visits a year (≥16 years of age) and is both a primary care facility (walk-in patients) and a tertiary referral centre for other hospitals in the area.

Cases were retrieved from the ED specific electronic patient chart database (E.care®) in which all clinical documentation (e.g., vital signs, laboratory results, notes, scanned copies handwritten records) is done by physicians and nurses. The search was performed by the IT-team of the ED using a comprehensive full-text search algorithm including all parts of the E.care® medical records. In brief, the automatic search identified all cases mentioning abuse, intoxication or related terms and a large number of substance names, including abbreviations and misspellings. The same sensitive search terms have been used in previous studies in Basel [6, 7] and are listed in the Additional file 1. By searching all parts of the medical records and using a wide spectrum of search terms we wanted to be sure that all relevant cases would be captured, even if that meant that a very large number of false positive cases would have to be reviewed manually and excluded (e.g., all cases with history of substance use including tobacco mentioned, even if not in relation to the presentation). The retrieved cases were reviewed by three of the authors of the study (two clinical fellows and one senior physician in clinical pharmacology and toxicology). A small proportion of not clear cases was reviewed together with the senior physician, who had experience in this field from previous studies in collaboration with Basel and the Euro-DEN project [5–7]. All cases included were also reviewed by the senior physician to asure that the same criteria were applied for all the cases. The charts of all cases were reviewed but only patients with acute toxicity were included. A recreational drug was defined as “a psychoactive compound that was taken for the purpose of recreational activities rather than for medical or work purposes or for self-harm”. The recreational drug(s) associated with the presentation were identified on the basis of one or a combination of the following: the patient’s self-reported use, information retrieved from witnesses, the opinion of the physician assessing the patient, and/or analytical results. Cases lacking substance self-report (e.g., because of coma, unwillingness to cooperate, language barriers), but with symptoms and/or analytical test typical of acute recreational drug toxicity were also included. Data abstracting was performed in a standardised manner [3, 5–7]. Exclusion criteria were: isolated ethanol intoxication, drug withdrawal and complications of chronic drug use (e.g., infected injection sites). Manifestations of drug withdrawal, although clinically relevant and in some cases potentially also life-threatening, were excluded as the main objective of the study was to investigate medical problems in relation to acute toxicity of psychoactive substances, which can differ greatly from the symptoms and management of withdrawal. Patients attending the ED requiring treatment because of symptoms and signs consistent with an alternate medical diagnosis and not primarily related to acute recreational drug toxicity (e.g., injury related presentation without signs of intoxication and subsequently found that the patient had used recreational drugs) were also excluded, as the presenting complaint was not related to the drug use, unless the trauma was directly related to drug use, e.g., as a result of hallucinations. The patient demographics (age, sex, hour and week day of ED visits), the substances used as reported by the patient or witnesses, the clinical effects, and clinical outcome were recorded. Clinical variables included the Glasgow Coma Scale (GCS) score, heart rate, blood pressure, respiratory rate, body temperature, laboratory tests and electrocardiography (ECG) findings. Hyperthermia was defined as a temperature ≥ 39 °C, measured by any method, hypothermia as a temperature < 35 °C, hypertension as systolic blood pressure ≥ 180 mmHg, hypotension as systolic blood pressure ≤ 90 mmHg. Hallucinations were defined as any deceptive or altered perception (visual, auditory, tactile, olfactory and/or gustatory), psychosis as any episode of delusions accompanied by confusion, hallucinations and lack of insight, seizures as any type of generalised tonic-clonic, myoclonic, partial or focal seizure. The severity of poisoning was assessed using the Poison Severity Score [8]. Minor toxicity refers to mild, transient and spontaneously resolving symptoms, moderate toxicity to pronounced or prolonged symptoms, and severe toxicity to severe or life-threatening symptoms. A urine drug screening test using an immunoassay (Triage® TOX Drug Screen, Alere, Cologne, Germany) was used to screen for amphetamines, barbiturates, benzodiazepines, cocaine, methadone, methamphetamines (including MDMA), opiates, phencyclidine (PCP), tricyclic antidepressants, and tetrahydrocannabinol (cannabis). The cut-off level was 1000 ng/mL for amphetamines, methamphetamines, and tricyclic antidepressants, 300 ng/mL for barbiturates, benzodiazepines, cocaine, methadone, and opiates, 50 ng/mL for cannabis, and 25 ng/mL for PCP [9]. In one case with suspected NPS use an additional liquid chromatography-mass spectrometry method was used. Ethanol blood levels were estimated from osmolar gap using the following equations: serum ethanol (g/L) = (serum osmolality - (2 * sodium (mEq/L) + glucose (mmol/L) + blood urea nitrogen (mmol/L))/ (1.25 * 21.71) and ethanol concentration (g/kg) = serum ethanol (g/L)/ (1.236) ‰ [10, 11].

## Results

Over the 4 year study period, from May 2012 to April 2016, there were 157,328 ED attendances. Among these, 73′258 were identified using the search algorithm as potential cases. However, most cases were excluded as the presentation was not related to acute drug toxicity. After applying the inclusion and exclusion criteria, 503 cases related to acute toxicity of substances used recreationally were detected in 408 different patients. Forty-five patients presented more than once to the ED due to symptoms/signs related to acute substance toxicity (27 patients twice, nine patients three times, five patients four times, one patient five times, two patients nine times, and one patient 16 times). The mean patient age was 33 years (range 16–74), and 68% were brought to the ED by ambulance. The patient characteristics are shown in Table 1.

**Table 1:** Patient characteristics

	Number of cases, *N* = 503 (%)
Male	344 (68)
Female	159 (32)
Age (years)	
16–20	67 (13)
21–30	169 (34)
31–40	136 (27)
41–50	89 (18)
> 50	42 (8)
Time of presentation	
Night arrival (20:00 – 8:00 h)	254 (50)
Weekend arrival (Friday 17:00 h - Monday 8:00 h)	233 (46)
Ethanol co-ingestion (self-reported)	
Yes	274 (54)
No	46 (9)
Not known	183 (36)
Drug use reported	
1 substance	260 (52)
> 1 substances	187 (37)
No drug use reported	13 (3)
No information available (e.g., coma, uncooperative)	43 (9)
Drug use analytical results	
1 substance	74 (15)
> 1 substances	178 (35)
Negative test (absence or insufficient test sensitivity)	25 (5)
No drug test performed	226 (45)

**Fig. 1:**
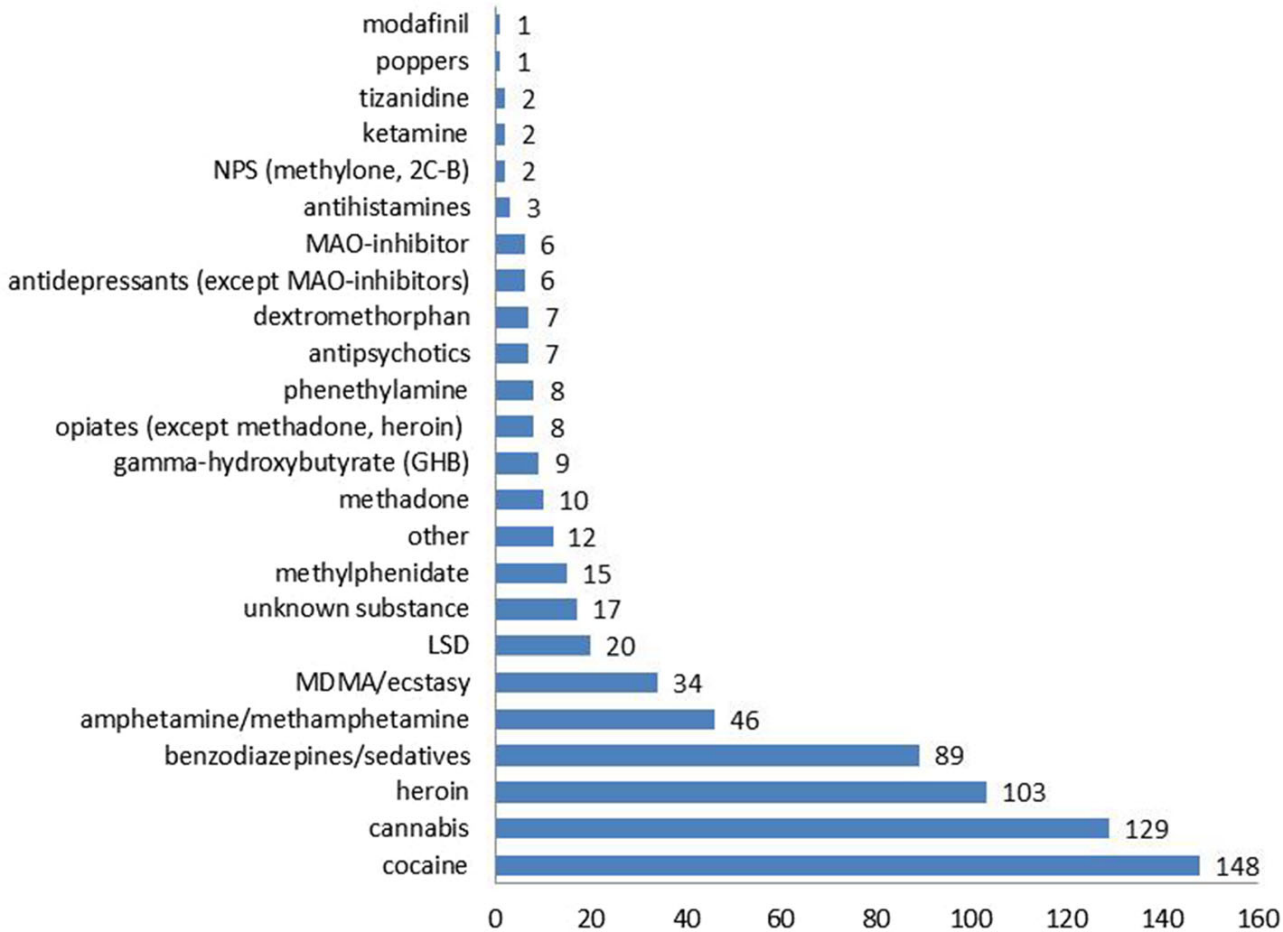
Reported substance use (count of cases)

The most commonly self-reported recreational drugs were cocaine (29%), cannabis (26%), heroin (20%) and benzodiazepines/sedatives (18%) (Fig. 1).

After exclusion of opioids and benzodiazepines, the most common recreationally used prescription drugs were methylphenidate (3%), antipsychotics (1%), dextromethorphan (1%), monoamine oxidase (MAO) inhibitors (1%), other antidepressants (1%), and antihistamines (0.6%). Substances classified as “other” included caffeine, “thai pills” (2 cases each), inhalation of methanol, nonsteroidal anti-inflammatory drugs (NSAIDs), melanin, pseudoephedrine, kamagra, propofol, “smileys”, and “stimulants” not further specified (1 case each). Three percent of the patients reported that they had used a substance without knowing what it was, in 9% of the cases no information was available on the substances taken, and 3% denied having used any drugs. These patients were included because they were judged by the assessing physician as being acutely intoxicated, on the basis of the symptoms and/or analytical results.

There were only two cases of NPSs: one with 3,4-methylenedioxymethylcathinone, a cathinone also known as methylone, and one with 2,5-dimethoxy-4(n)-propylphenethylamine (2C-P) sold as 2,5-dimethoxy-4-bromophenethylamine (2C-B) [12], both of moderate severity. The characteristics of those cases are shown in Table 2.

**Table 2:** NPS cases characteristics

NPS self-reported	Age/ gender	NPS dose self-reported	Co-ingestion alcohol	Clinical presentation	Treatment	Tox. laboratory analysis	Outcome
Methylone	32/ female	1 - 1.5 g cumulative during 6.5 h	yes	Palpitations, fear, agitation, sweating, tremor, vertigo, paraesthesias, muscle twitching, vomiting	1 mg sublingual lorazepam 10 mg i.v. metoclopramide intravenous fluid administration	Negative (immunoassay)	Discharged home the same day
2C-B	19/ male	25 mg	yes	Severe hallucinations, mydriasis, tachycardia, agitation, confusion	benzodiazepines 4 mg i.v. haloperidol	2C-P (liquid chromatography-mass spectrometry)	Discharged home 11 h later

**Fig. 2:**
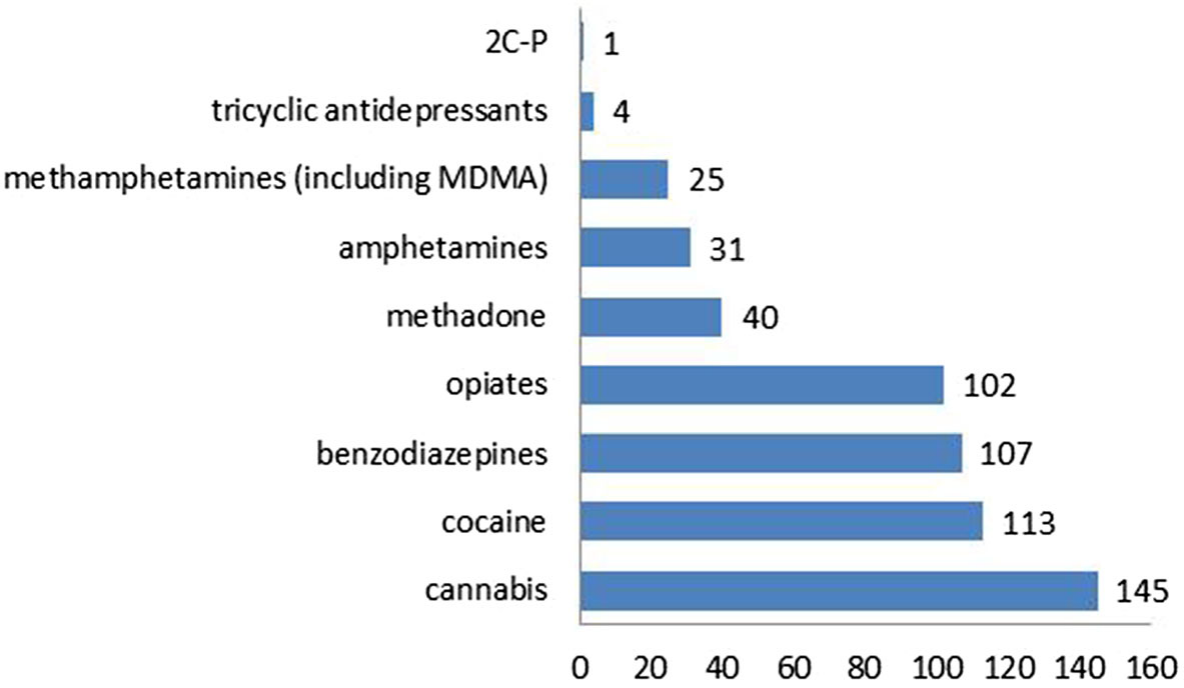
Substances analytically detected (count of cases)

Overall, a drug screening was available in 277 cases (55%). The most frequently detected substances were cannabis (29%), cocaine (22%), benzodiazepines (21%) and opiates excluding methadone (20%) (Fig. 2).

Alcohol co-use was analytically confirmed in 35% of the cases. Among the 175 cases with a positive ethanol test, the median alcohol concentration was 1.4‰ (range 0.01–4.7).

Table 3 summarises the medical problems.

**Table 3:** Clinical characteristics of acute recreational drug intoxications

	Number of cases, *N* = 503 (%)
Cardiovascular	
Chest pain	34 (7)
Palpitations	41 (8)
Dyspnea	25 (5)
Hypertension (systolic blood pressure ≥180 mmHg)	14 (3)
Hypotension (systolic blood pressure ≤90 mmHg)	18 (4)
Tachycardia (>100 beats per minute)	144 (29)
Myocardial infarction	3 (<1)
Arrhythmias	5 (1)
Psychiatric	
Anxiety, nervousness, and/or fear	121 (24)
Psychosis	71 (14)
Hallucinations	54 (11)
Agitation and/or aggression	179 (36)
Panic attack	7 (1)
Insomnia	18 (4)
Suicidal ideation	14 (3)
Neurological	
Loss of consciousness (GCS < 8) on presentation or pre-hospital	107 (21)
Impaired consciousness (GCS <15) on presentation or pre-hospital	291 (58)
Vertigo and/or dizziness	39 (8)
Headache	24 (5)
Paraesthesias	17 (3)
Seizure	26 (5)
Tremor	22 (4)
Amnesia	33 (7)
Other neurological symptoms	36 (7)
Miosis	87 (17)
Mydriasis	74 (15)
Respiratory depression	69 (14)
Intracranial haemorrhage	2 (<1)
Miscellaneous	
Hyperventilation	22 (4)
Nausea and/or vomiting	61 (12)
Sweating	19 (4)
Malaise	13 (3)
Abdominal pain	14 (3)
Hyperthermia (≥39.0 °C)	3 (<1)
Hypothermia (<35.0 °C)	10 (2)
Muscle cramps	20 (4)
Injuries (e.g., fracture, wound)	23 (5)
Acute kidney injury	2 (<1)
Elevated creatine kinase (>250 U/L)	71 (14)
Weakness, walking impairment	20 (4)

Among all 503 cases, 21% presented with severe intoxication (Table 4) and there were two fatalities: a 39-year old female patient with cerebral hypoxia after intake of approximately 180 mg methadone from the substitution medication of her partner, and a 33-year old male patient with cerebral infarction and analytical detection of cocaine and cannabis (no self-report available (GCS 5 pre-hospital, intubated at presentation), diagnosed as possible cardioembolic complication in the context of cocaine use).

**Table 4:** Severity of poisoning and outcome

	Number of cases, *N* = 503 (%)
Severity of poisoning	
Minor	177 (35)
Moderate	220 (44)
Severe	104 (21)
Fatal	2 (<1)
Outcome	
Medically discharged home	299 (59)
Self-discharged	42 (8)
Admission to critical care unit	32 (6)
Admission to ward other than critical care unit	19 (4)
Admission to psychiatric clinic	111 (22)

Severe complications included 3 acute myocardial infarctions (following cocaine use by a 45-year old male patient, cannabis use by a 52-year old male patient, and gamma-hydroxybutyrate (GHB) and kamagra use by a 50-year old male patient), 2 intracranial haemorrhages (43-year and 48-year old male patients, cocaine self-reported in one case and in the other case analytically detected together with cannabis and opiates), as well as psychosis and seizures in 71 and 26 cases, respectively (Table 3). The outcome data are shown in Table 4. In 374 cases (74%) medical treatment including oxygen and intravenous fluid administration was provided. Tracheal intubation was performed in 16 cases (3%). Sedating drugs (i.e., benzodiazepines and/or antipsychotics) were administered in 128 cases (25%), an antidote (i.e., naloxone, flumazenil, activated charcoal and/or biperiden) was given in 54 cases (11%).

## Discussion

The present study described the presentations related to acute recreational drug toxicity at a large urban ED in Bern, Switzerland. Over a period of four years (May 2012 - April 2016), most presentations due to acute recreational drug toxicity were associated with cocaine and cannabis use. Those were also the substances most commonly analytically detected. Only 2 presentations were related to acute toxicity of NPSs. The typical patient was young and male, and alcohol had been co-ingested in the majority of the cases. There were two fatalities and further severe complications, such as myocardial infarction and intracranial haemorrhage, while approximately one fifth of the cases were unconscious at presentation or prior to hospital admission. However, most intoxications were of moderate severity and most patients were discharged home directly from the ED.

Comparing our findings with the similar studies from Basel [6, 7], another city in Switzerland with an University Hospital and a large urban ED, in both centres cannabis and cocaine seem to be the leading substances resulting in ED presentations due to acute toxicity, possibly indicating a general trend across the country. In the European data, cocaine and cannabis were also among the most commonly reported substances (number 2 and 3, respectively), but the most commonly reported drug was heroin [5]. Possible reasons for such differences could be local trends, the well-established heroin substitution programs in Switzerland, and price differences of substances among countries. In all studies, the typical patient presenting to the ED due to acute toxicity of recreational substances was male, in his early thirties, and was usually brought to the ED by ambulance overnight after using a classical recreational substance, often in combination with alcohol [5–7].

Another interesting similarity among the studies mentioned is that - despite the dramatic increase in the number of NPSs detected in recent years - presentations related to NPSs were much less common than those related to classical recreational drugs. This may indicate that NPSs possess less acute toxicity than classical recreational substances, that NPSs are consumed to a lesser extent or may be due to the fact that they escape detection with the normal immunoassay in many cases, especially if the patient does not report or is unaware of their use (e.g., if they are sold under other names, or in combination with other substances). One such example in the present study was the 2C-P case (detected by using liquid chromatography-mass spectrometry, usual doses 6–10 mg), which was mistaken for the better-known, less potent and shorter-acting 2C-B (usual doses 10–20 mg), and thus led to more severe and prolonged intoxication after intake of approximately 25 mg [12]. The 2C drugs are a subgroup of phenethylamines with primarily hallucinogenic properties [13]. Different ligands at position 4 on the phenyl ring within this family (e.g., bromine in 2C-B or propyl in 2C-P) can lead to great differences in receptor affinity [14]. The second NPS reported in our study was the synthetic cathinone 3,4-methylenedioxymethylcathinone (methylone or βk- MDMA) [15]. In this case, the patient reported buying the substance from Beijing, where it was still legal. The immunoassay drug screening was negative, as this test cannot detect most NPSs, so no NPS would have been registered without self-report. Methylone is a first generation synthetic substance from the group of the cathinones [16], which are derivatives of the naturally occurring beta-ketone amphetamine analogue found in the leaves of the Catha edulis plant [17]. Methylone, the beta-ketone analogue of MDMA, emerged in the recreational drug market in the mid-2000’s under the brand name “Explosion” and was the first of these substances to be marketed via the Internet and at smart shops [16]. Desired effects range from amphetamine-like stimulation (e.g., increased energy, alertness) to entactogenic effects similar to those produced by MDMA (e.g., empathy, openness) [15]. In accordance with the symptoms reported by the patient in our study, the most common adverse effects include palpitations, vomiting, anxiety, sweating, unsteadiness of the hands, but also seizures, hyperthermia, psychosis, hallucinations and suicidal ideation [15]. Fatalities have also been reported [18–20]. Common oral doses (the most popular route of administration) are 100–300 mg, and doses higher than 250 mg are considered as “heavy” consumption [16]. The total dose reported by our patient (1–1.5 g) is thus very high. However, it is possible that the patient used a larger dose as “boosting” first, followed by smaller doses (“bumps”) to maintain the desired effects [15].

Beside the use of classical recreational drugs and NPSs, a further issue of increasing concern is the recreational use of prescription drugs. Among the ten cases of reported recreational methadone use in our study, seven were in a methadone substitution program, and in one of the fatal cases the patient used the methadone of her partner who was under maintenance therapy. Such cases demonstrate some of the risks related to long-term opioid maintenance treatment and the importance of the adjunctive psychosocial care of these patients. Sedatives and hypnotics are after opioids the most commonly misused prescription drugs in Europe [21]. Accordingly, benzodiazepines were the more commonly self-reported substances after cocaine, cannabis and heroin in our study. According to surveys, the main reasons for their use were to help sleep, to cope with stress, and/or to “get high” and almost 15% of the participants misusing them reported doing so at least once weekly [22]. Although the abuse and misuse potential of many of the other self-reported recreationally used prescription drugs in our study is known and plausible on the basis of their mechanism of action (e.g., psychostimulant effect of methylphenidate and modafinil, dissociative effect of N-methyl-D-aspartate (NMDA) receptor antagonists such as ketamine and dextromethorphan, sedative effect of antihistamines), this is not always the case for other substance groups, e.g., antidepressants. Antidepressants include a variety of substances with different pharmacological properties (e.g., sedation, stimulation), some of which make abuse attractive [23]. For example, bupropion, the most commonly misused antidepressant of the last decade [23], acts by inhibiting norepinephrine and dopamine reuptake [24], similarly to other indirect sympathomimetics (e.g., cocaine). In our study, recreational bupropion was administered intravenously in one case (approximately 1500 mg i.v.) and was intended to cause an amphetamine-like effect. Intravenous abuse of bupropion is also found in case reports and the drug can be acquired relative easily by expressing the wish to quit smoking [25]. Another category of antidepressants commonly misused are MAO inhibitors, which prevent the breakdown of monoamine neurotransmitters by MAO-A and/or MAO-B [23]. In our study, MAO inhibitor use was reported in six cases by the same patient, who presented a total of 16 times to the ED due to acute recreational substance use toxicity. The patient used the MAO inhibitor in combination with phenethylamine in five of the cases, most probably as an attempt to decrease its breakdown and thus potentiate its effects. Phenethylamine, also known as β-phenylethylamine, enhances catecholamine release, and can be obtained legally as dietary supplement. Unlike its derivatives (e.g., amphetamine), it is rapidly metabolised primarily by MAO-B, thus normally preventing significant concentrations from reaching the brain [26, 27]. Recreational use of methylphenidate was reported in 15 cases in our study by 12 different patients (one patient presented four times). An increase in reports of non-medical use of methylphenidate in recent years has been shown in a retrospective analysis from the Swiss Toxicological Information Centre [28]. In that study, the most common route of application was oral, but nasal and intravenous administration route was used in a substantial proportion of the cases, and 40% of the abusers reportedly used methylphenidate as a prescription drug for ADHD. Similar to those findings, half of the patients in our study (6 out of 12) received methylphenidate as treatment for their ADHD, but used it differently than prescribed (higher doses and/or other route of administration), and one patient took the drug from his girlfriend who had an ADHD diagnosis. In our study, the reported administration route was oral in the majority of the cases (9/15), i.v. and nasal administration were reported in 2 and 1 case(s), respectively, while in 3 cases the route of administration was not specified. Methylphenidate was also the most commonly reported prescription drug used for neuroenhancement (e.g., nonmedical use of a substance to improve cognitive function) in surveys among Swiss university students [29] and Swiss secondary school students [30].

As main limitations of the study, there were some missing data (the initial patient data were not recorded in a standardised manner), some symptoms described could have been related to substance withdrawal and not acute toxicity (e.g., seizures associated with benzodiazepine or alcohol withdrawal) or to aetiologies other than the acute toxicity of the psychoactive substances studied (e.g., vomiting because of alcohol co-ingestion or gastroenteritis, pre-existing psychiatric pathologies not recognised or documented as such). Because of the co-use of alcohol leading to impaired consciousness in many cases, severe intoxications may have been overrepresented. Furthermore, substances taken as medication (e.g., benzodiazepines, methadone, antidepressants) or given by the paramedics (e.g., benzodiazepines) could have been overrepresented in the analytical results; this also applies to substances that can be detected in urine samples beyond the time period of acute intoxication (e.g., cannabis). On the other hand, substances which can be detected only during a very short time period (e.g., GHB) or cannot be detected with the immunoassay test used in the study (e.g., LSD, NPSs) may have gone undetected if their use was not reported. Additionally, some patients with psychiatric symptoms were transferred to a psychiatric ward shortly after arrival at the ED, thus not allowing full documentation of treatment and outcome. Moreover, data from only one ED may reflect local trends and may not be representative.

The strengths of our study include the sensitive search algorithm used and the detailed documentation, in contrast to studies based on coded diagnoses only, which would have led to missing relevant cases with substance use not mentioned as a diagnosis, or studies based on laboratory data only, which would have caused the inclusion of not clinically relevant cases (e.g., cannabis detection long after acute intoxication). Further strengths include the drug screening performed in most cases, the relatively long time period studied, and the collection and analysis of data in a systematic manner, which allowed a comparison with the similar data from other cities in Switzerland (i.e., Basel) and elsewhere in Europe.

## Conclusion

In conclusion, this retrospective study at a large urban ED in Bern, Switzerland over a period of 4 years showed that most presentations due to acute recreational drug toxicity were associated with cocaine and cannabis use and were mainly characterised by central nervous system depression, sympathomimetic toxicity and/or psychiatric disorders. Those results are in accordance with the-currently only limited- data available from other parts of Switzerland (Basel), thus potentially indicating national trends. Beside classical recreational drugs, the substances most commonly reported in this study were prescription drugs, whereas ED presentations related to acute toxicities of NPSs appear to be uncommon, either because NPSs are not commonly used, or because their use leads to ED presentations less often. Future research from other parts of Switzerland, but also Europe, could provide more evidence to help distinguish between these two hypotheses. Furthermore, the data of this study can contribute to the recognition of prescription drug abuse potential and to the development of preventive strategies such as clinician training to ensure appropriate prescription, and regulation of substances with abuse potential.

## Additional file

Additional file 1: Search terms. (DOCX 14 kb)

## Abbreviations

2C-B: 2,5-dimethoxy-4-bromophenethylamine
2C-P: 2, 5-dimethoxy-4(n)-propylphenethylamine
ADHD: Attention deficit hyperactivity disorder
ECG: Electrocardiography
ED: Emergency department
EMCDDA: European Monitoring Centre for Drugs and Drug Addiction
Euro-DEN: European Drug Emergencies Network
GCS: Glasgow Coma Scale
GHB: Gamma-hydroxybutyrate
MAO: Monoamine oxidase
MDMA: 3,4-methylenedioxymethamphetamine
methylone: 3,4-methylenedioxymethylcathinone
NMDA: N-methyl-D-aspartate
NPS: Novel psychoactive substance
NSAIDs: Nonsteroidal anti-inflammatory drugs
PCP: Phencyclidine
