# Supplementary material for: Presentations to an urban emergency department in Bern, Switzerland associated with acute recreational drug toxicity
Source: Scand J Trauma Resusc Emerg Med. 2017 Mar 7;25:26. doi: 10.1186/s13049-017-0369-x (PMC5340017; doi:10.1186/s13049-017-0369-x)
Supplement: Additional file 1: — Search terms. (DOCX 14 kb) [file 13049_2017_369_MOESM1_ESM.docx]

**Search terms**:

F14.0 Kokainintoxikation, F15.0 Amphetaminintoxikation, F16.0 Halluzinogenintoxikation, F19 Substanzmissbrauch, F19.0, Intoxikation, F15 Amphetamin, F16 Halluzinogen, Intoxikation, Vergiftung, Missbrauch, Abusus, Mischintoxikation, Einnahme, Polytoxikomanie, Drogenpsychose, Abhängigkeit, overdose, Überdosierung, Ingestion, Kokain, Kocain, Cokain, Cocain, Crack, Coca, Freebase, speedball, Heroin, Opiat, Methadon, Codein, dextromethorphan, Cannabis, Hash, THC, Amphetamin, Speed, Methamphetamin, Crystal, crystal, meth, yaba, Ecstasy, MDMA, tasy, entaktogen, entactogen, , GHB, liquid extasy, tasy, liquid, gamma, hydroxy, butyrat, Buttersäure, GBL, butandiol, , Spice, JWH, cannabinoid, synthetisch, Kräutermischung, Pilze, Psilocybin, Halluzinogen, LSD, 2-CB, 2-CD, 2-CE, 2-CI, mescalin, Droge, Designer Droge, Badesalz, research chemical, cathinon, kathinon, katinon, legal high, stimulans, Phenethylamin, DOI, DOM, DOB, NBoM, angel dust, PCP, phencylidin, Meo-PCP, Mephedron, Naphyron, Methylon, Ethylon, Butylon, MDEA, MBDB, MDA, MDPV, PMA, PMMA, MDAI, 2C, 2D, MEC, Amfepramon, metamfepramon, flephedron, MDPBP, Fluoroamphetamin, MTA, Methedron, AI, Tryptamin, DMT, Salvinorin, Salvia, Piperazin, mCPP, TFMPP, BZP, DBZP, pCPP, 4-FPP, Phenylpiperazin, Benzylpiperazin, Pipradrol, D2PM, Bromo Fly, Fly, AMT, DXM, DPH, DHM, methexetamine, MXE, Lachgas, Amylnitrit, poppers, Stechaphel, Tee, Engelstrompete, Bilsenkraut, Tollkirsche, Ketamin, MPH, Ritalin, Benzodiazepin, benzo
